# Cervical Paratracheal Bronchogenic Cyst Mimicking Nodal Metastatic Disease in a Colorectal Cancer Patient: Diagnostic Utility of Neck Ultrasound-Guided Fine Needle Aspiration

**DOI:** 10.7759/cureus.90409

**Published:** 2025-08-18

**Authors:** Christien-Jorde Dacres, Yousef Shweihat, John Diks

**Affiliations:** 1 Pathology, Marshall University Joan C. Edwards School of Medicine, Huntington, USA; 2 Internal Medicine, Marshall University Joan C. Edwards School of Medicine, Huntington, USA

**Keywords:** bronchogenic cyst, cervical cyst, colorectal cancer surveillance, cytopathology, metastasis mimic, ultrasound-guided fna

## Abstract

Bronchogenic cysts are rare congenital lesions, typically identified in childhood but occasionally found in adults as incidental findings on imaging. In oncology patients, these lesions can present a diagnostic challenge due to their potential to mimic metastatic disease. We report a case of a 78-year-old woman undergoing routine surveillance for Stage II colorectal cancer who was found to have a paratracheal cystic lesion suspicious for malignancy. Ultrasound-guided fine needle aspiration (US-FNA) targeted posterior to the carotid artery and aspirated necrotic material, yielding pathognomonic ciliated epithelium without malignancy. Coupled with absent FDG avidity on PET and negative cultures, cytology confirmed a benign bronchogenic cyst, excluding metastasis and preventing unnecessary treatment. This case underscores that cervical bronchogenic cysts are critical malignancy mimics in oncology patients and demonstrates US-FNA’s unique utility in obtaining contamination-free diagnostic samples, overcoming limitations of other approaches, to avert intervention risks during cancer surveillance.

## Introduction

Bronchogenic cysts (BCs) are rare congenital malformations first described in 1859, originating from abnormal budding of the embryonic tracheobronchial tree (foregut-derived) during early gestation [1]. Their reported incidence is approximately 1 per 42,000-68,000 hospital admissions [2]. A recent case of thoracoscopic excision highlights their typical presentation in the thorax [3]. BCs constitute 10-15% of mediastinal tumors and 50-60% of mediastinal cystic lesions [2,4-6], with a male predominance (66.7% in pediatric series) [7-9]. Diagnosis occurs across all age groups, showing bimodal peaks in early childhood (<5 years) and adulthood (20-40 years) [4,7-10]. In a 20-year retrospective study, 51.1% of bronchogenic cysts were located in the subcarinal area, and 31.1% were paratracheal. Notably, 20% of these cysts were detected incidentally during imaging studies, underscoring the asymptomatic nature of many cases [8]. While most (>50%) are thoracic (subcarinal/paratracheal) [6,8,10,11], ectopic development along the foregut enables presentations above/below the diaphragm [12]. Cervical BCs are exceptionally rare (<5% of cases) [10,13], often mimicking thyroglossal duct cysts, branchial cleft cysts, hydatid cysts, or metastatic nodes in cancer patients [6,14-16]. 

Preoperative diagnosis remains challenging due to nonspecific CT/MRI features (e.g., homogeneous fluid density) [2,5,6,11,15]. For cervical lesions, ultrasound-guided FNA (US-FNA) is the diagnostic cornerstone [17,18]. When performed per standardized protocols, it enables contamination-free sampling of cyst wall material - a critical advantage over EBUS-guided FNA, which carries >40% contamination risk from respiratory epithelium. Such contamination causes diagnostic ambiguity, while US-FNA's extra-airway approach yields pristine specimens (ciliated epithelium + mucinous debris), improving diagnostic accuracy. This prevents unnecessary surgeries and staging errors in cancer patients [14,17-19]. We report a rare cervical bronchogenic cyst in a patient with prior colon adenocarcinoma, emphasizing how US-FNA helped prevent misdiagnosis and unwarranted treatment.

## Case presentation

**Figure 1:**
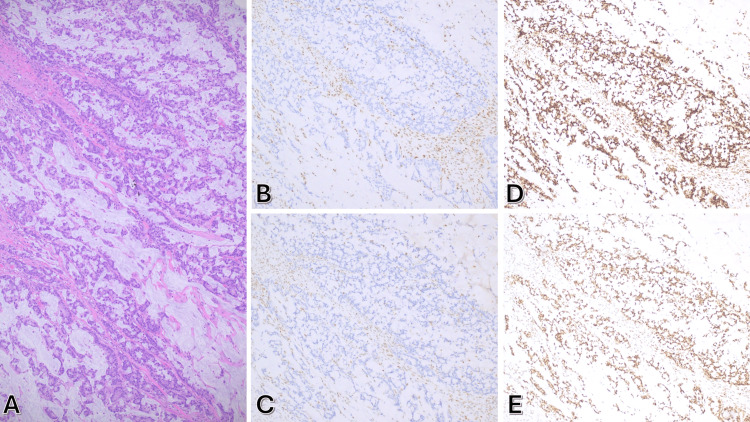
(A) Hematoxylin and Eosin (H&E) showing invasive mucinous adenocarcinoma of the colon. (B) & (C) MLH1 and PMS2 IHC showing loss of nuclear staining while intact stanning of internal control. (D) & (E) MSH2 and MSH6 IHC showing intact nuclear staining.

A 78-year-old woman with a history of Stage II (T3N0M0) (Figure 1A) right-sided colon adenocarcinoma underwent curative right hemicolectomy in 2021. Surgical pathology confirmed negative margins with 0/32 lymph nodes involved. Immunohistochemical profiling revealed mismatch repair deficiency (loss of MLH1 and PMS2 nuclear expression; intact MSH2/MSH6) (Figure 1B-1E) and a BRAF V600E mutation. Given the absence of high-risk features (no obstruction/perforation, margin-negative resection), adjuvant chemotherapy was deferred per National Comprehensive Cancer Network (NCCN) guidelines [20]. She entered structured surveillance involving serial computed tomography (CT) scans, serum carcinoembryonic antigen (CEA) monitoring, and colonoscopies. 

**Figure 2:**
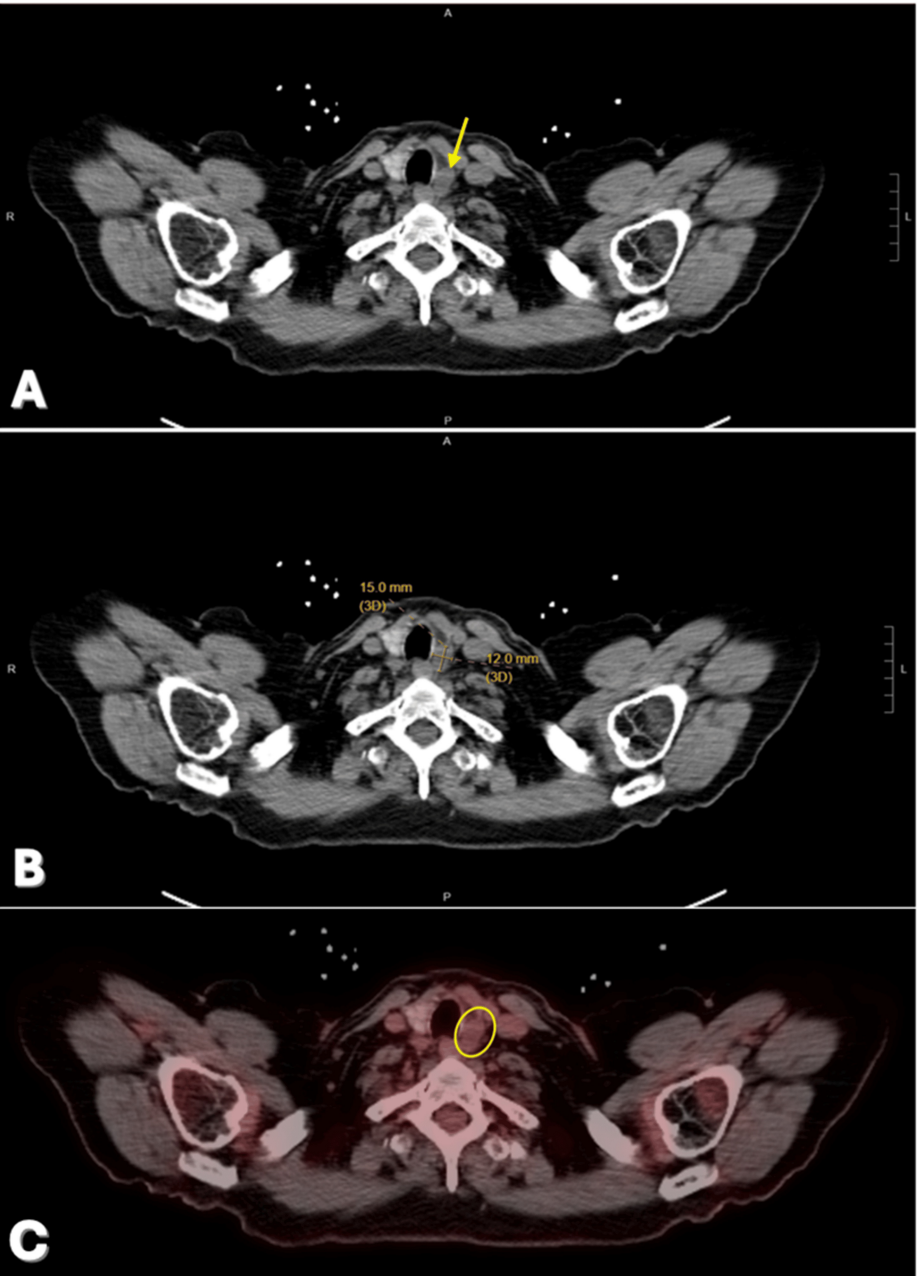
(A) A Chest CT scan of left Paratracheal Lesion. The lesion appeared adjacent to the left thyroid lobe (arrow). (B) Anterior position of the patient identifying a 1.5 × 1.2 cm left paratracheal lesion suggestive of nodal metastasis. (C) A positron emission tomography (PET) and computed tomography (CT) (PET/CT) scan of left Paratracheal Lesion demonstrated no FDG avidity (SUV max 1.2) (circle). A: Anterior, P: Posterior, L: Left, R: Right

Surveillance remained unremarkable until September 2024, when a routine chest CT incidentally identified an asymptomatic new 1.5 × 1.2 cm left paratracheal cystic lesion adjacent to the thyroid lobe with interval enlargement (Figure 2A-2B), radiologically concerning for lymphoproliferative disease or metastatic lymphadenopathy. Subsequent neck ultrasound localized the lesion suprasternally, posterior to the left carotid artery. Ultrasound-guided fine-needle aspiration (US-FNA) under local anesthesia and sterile technique aspirated thick, yellow necrotic fluid. Specimens were sent for cytopathology and comprehensive microbiology (aerobic/anaerobic bacterial, fungal, and acid-fast bacilli cultures). Positron emission tomography (PET) demonstrated no FDG avidity (SUV max 1.2) (Figure 2C). Serum CEA level was 6.4 ng/mL (Reference range for smokers is less than or equal to 3 ng/ml).

**Figure 3:**
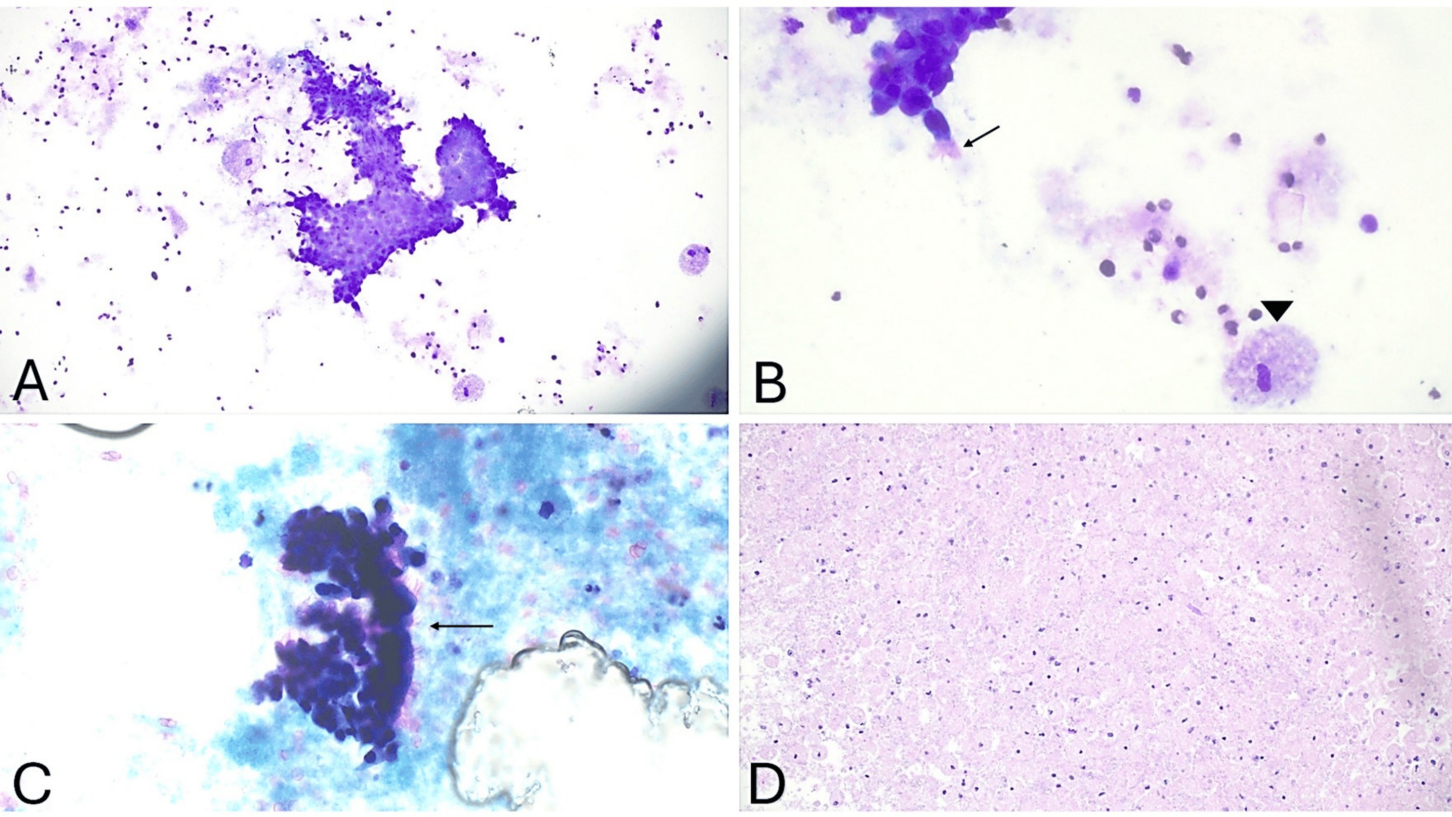
(A) Diff-Quik stain (x200) presents a cluster of ciliated columnar epithelial cells in a background of grungy necrotic debris. (B) Diff-Quik stain (x600) shows ciliated columnar epithelium (arrow) and macrophages (arrowhead). (C) PAP stain (x600) shows a cluster of ciliated columnar epithelium (arrow). (D) Hematoxylin and Eosin (H&E) cell block (x200) shows sheets of macrophages with necrotic debris background. No dysplasia or malignancy was identified.

Cytologic evaluation revealed predominantly macrophages with clusters of ciliated columnar epithelium in a background of grungy proteinaceous debris, with no malignant cells identified (Figure 3). Polarized microscopy was negative for crystals. Microbiological cultures returned negative after a six-week incubation period. These findings confirmed a bronchogenic cyst, excluding metastatic adenocarcinoma, infectious processes (e.g., tuberculous lymphadenitis), and salivary gland neoplasms (e.g., Warthin tumor). 

At the seven-month follow-up (April 2025), the patient remains asymptomatic with stable imaging. She continues NCCN-recommended surveillance without intervention for the cyst. 

## Discussion

Bronchogenic cysts are recapitulation of bronchial structure. Histologically, they are lined by ciliated columnar epithelium and often contain smooth muscle, mucous glands, and occasionally cartilage or dystrophic calcifications [1,5]. These cysts may communicate with the airway, although this is not always present. In our case, cytology revealed rare ciliated columnar epithelial cells, necrotic debris, and abundant macrophages-findings that are consistent with a bronchogenic cyst and not characteristic of other cystic lesions. 

Most of BCs are diagnosed incidentally on imaging. In the setting of active cancer surveillance, distinguishing them from metastatic disease is critical to avoid overtreatment. Accurate cytologic diagnosis of a bronchogenic cyst requires careful distinction from other cystic lesions of the mediastinum and lung [1,2]. The most important differentials include esophageal duplication cysts, thymic cysts, cystic teratomas, pulmonary sequestration, congenital cystic adenomatous malformation (CCAM), branchial cleft cysts, thyroglossal duct cysts, hydatid cysts, lymphangiomas, and dermoid cysts [5]. Esophageal duplication cysts can present similarly but are typically lined with both ciliated epithelium and gastrointestinal-type mucosa and show a double muscular layer, which was not observed in this case [1,4,15]. Thymic cysts, located in the anterior mediastinum, originate from remnants of thymic tissue and often contain thymic epithelium or Hassall's corpuscles, neither of which was present in our sample. Cystic teratomas may contain elements from all three germ layers, including neural, glandular, and squamous tissues, which were absent. Pulmonary sequestration refers to a non-functioning segment of lung tissue without an airway connection and typically has a systemic arterial supply, a feature that can be identified radiologically but was not noted here [5]. CCAM presents as a cystic or solid lesion in neonates or infants, lined by bronchiolar epithelium and lacking mature alveoli. Our patient’s age and imaging findings exclude this entity [5]. Branchial cleft cysts, although sometimes ciliated, typically have lymphoid tissue in the cyst wall and are usually located in the lateral neck [6,10,15]. Thyroglossal duct cysts occur in the midline neck and move with swallowing, which was not the case here [6,10,15]. Lymphangiomas are benign lymphatic malformations that appear as multiloculated cystic lesions, usually in children [5,8,15]. Dermoid cysts, composed of skin appendages and ectodermal elements, typically occur in specific anatomic regions and show keratin debris or squamous cells on cytology, which were not present [5]. Hydatid cysts, though uncommon, are caused by Echinococcus species. They show distinct imaging features and need specific serologic testing [16]. Considering the lesion’s location in the paratracheal region, cytological presence of ciliated columnar cells, lack of germ cell or gastrointestinal components, negative infectious workup, and absence of lymphoid or squamous elements, the findings support a bronchogenic cyst as the most consistent diagnosis (Table 1).

**Table 1:** This table aligns clinical, histologic, and imaging features to help differentiate these cystic lesions in the neck and mediastinum. Data adapted from multiple sources, including Cuypers et al., 1996 [1], Takeda et al., 2003 [4], McAdams et al., 2000 [5], Jeung et al., 2002 [6], Kieran et al., 2010 [15], Salamone et al., 2016 [16], with in-text citations corresponding to the reference list. CCAM: congenital cystic adenomatous malformation

Diagnosis	Typical Location	Cytological and Histologic Features	Imaging/Clinical Clues	Key Distinguishing Points
Bronchogenic cyst [1]	Mediastinum (subcarinal, paratracheal), cervical (rare)	Ciliated columnar epithelium, smooth muscle, mucous glands, possible cartilage	Homogeneous cystic lesion; asymptomatic/incidental	Ciliated cells in cytology; no GI mucosa; contamination-free FNA specimen
Esophageal duplication cyst [5,15]	Posterior mediastinum	Ciliated + gastrointestinal mucosa, double muscular layer	Posterior mediastinal cystic lesion	Double muscle layer; GI mucosa presence
Thymiccyst [4-6]	Anterior mediastinum	Thymic epithelium, Hassall’s corpuscles	Anterior mediastinal cyst	Presence of thymic tissue or Hassall’s corpuscles
Cystic teratoma [4-6]	Mediastinum, gonads	Tissues from all three germ layers	Mixed cystic-solid mass with varied tissue types	Germ layer derivatives (neural, glandular, squamous)
Pulmonary sequestration [4]	Lung parenchyma	Non-functioning lung tissue without airway	Systemic arterial supply on imaging	Systemic artery feeding; lack of airway connection
CCAM [4]	Neonatal lung	Bronchiolar epithelium, no mature alveoli	Cystic/solid lung lesion in infants	Neonatal presentation; bronchiolar epithelium
Branchial cleft cyst [15]	Lateral neck	Ciliated epithelium + lymphoid tissue	Lateral neck cystic mass	Lymphoid tissue in wall; lateral neck location
Thyroglossal duct cyst [15]	Midline neck	Respiratory or squamous epithelium	Midline neck cyst moving with swallowing	Midline location; moves on swallowing
Lymphangioma [15]	Neck, axilla, mediastinum	Dilated lymphatic channels	Multiloculated cystic lesion; usually in children	Multiloculated; pediatric age group
Dermoid cyst [15]	Midline/paramedian areas	Skin appendages, keratin debris, squamous cells	Cystic lesion with fat or calcifications	Keratin, hair, skin elements in cytology
Hydatid cyst [16]	Liver, lungs, sometimes neck	Laminated membranes, hooklets (Echinococcus)	Characteristic imaging (daughter cysts)	Positive serology; specific imaging features

Although no direct comparative studies have assessed the diagnostic yield or invasiveness of US-FNA versus EBUS-FNA, particularly for lesions in the cervical region, each modality has been independently studied [17-19]. Ultrasound-guided fine-needle aspiration (US-FNA) is preferred for evaluating superficial, easily accessible neck lesions such as thyroid nodules and cervical lymph nodes. It allows real-time imaging guidance for precise needle placement, facilitates targeted sampling of cyst walls, and benefits from on-site cytology, which improves specimen adequacy and immediate verification of key diagnostic elements like ciliated epithelium and mucin debris. This method is especially effective for diagnosing cervical and paratracheal bronchogenic cysts and helps reduce false negatives and unnecessary surgeries [17,18]. In contrast, endobronchial ultrasound-guided FNA (EBUS-FNA) is more invasive and typically reserved for deeper, more complex, centrally located lesions such as mediastinal or hilar lymph nodes, or when US-FNA results are inconclusive. EBUS-FNA has shown strong diagnostic performance in benign conditions like sarcoidosis and tuberculosis, but remains less studied in lesions at the superior thoracic inlet, where continuity between cervical and mediastinal structures can complicate interpretation. At this junction, epithelial contamination and overlapping anatomy may affect diagnostic accuracy, making anatomic precision essential during EBUS-FNA procedures [19].

## Conclusions

This case illustrates two fundamental principles: First, the anatomic plasticity of foregut-derived cysts permits migration along embryonic pathways, accounting for cervical manifestations. Second, in cancer patients with cystic cervical lesions, US-FNA should constitute the initial diagnostic modality. Its capacity to retrieve pathognomonic cytology while avoiding false-positive interpretations remains unparalleled. When coupled with metabolic imaging, this approach prevents hazardous overtreatment in oncology cohorts. 
